# Sir John Furley

**Published:** 1905-06-24

**Authors:** 


### SIR JOHN FURLEY.

Almost coincidently with the appearance of the third volume of the *Times* "History of the War in South Africa," and with its gruesome recalling to mind of the degree in which the bloodshed and pestilence attendant upon the great struggle were aggravated by national and official unwillingness to recognise either its magnitude or the character of the repressive action for which it called, we have to welcome a publication of a widely different character, in a sketch by himself of the career of the great leader of what may perhaps be described as military philanthropy, the bearer of the Geneva Cross in many countries and through many campaigns, Sir John Furley. In one sense the word "bearer" is inappropriate; for he never actually wore the brassard on duty, on account of the manner in which it had been dragged through the dirt by unauthorised people, newspaper correspondents, commercial travellers, tourists, and possibly even by spies. But he has been the most active and most distinguished embodiment of his period of all that the Cross was originally intended to imply; and he has also rendered valuable help in co-ordinating for the benefit of the sick and wounded on both sides of all recent European warfare, not only the efforts of the Red Cross Societies of the several countries concerned, but also those of the Order of the Hospital of St. John of Jerusalem in England, the descendant and representative of the Knights Hospitallers of the Crusades, an order of which he has for many years been an active and highly distinguished member. The volume, entitled "In Peace and War," which Sir John has just issued, may be described as a sort of official autobiography, which, while imparting only too little information about the man himself, contains something like a history of his official career, from the commencement of the war between Germany and Denmark in 1864 to the close of that in South Africa. By far the largest part of the book is devoted to the Franco-German war of 1870-71, and to the subsequent devastation of Paris by the Commune, occurrences during which Sir John was omnipresent, as indifferent to personal danger as a Marlborough or a William the Third, constantly arrested or detained by suspicious persons on both sides, sometimes compelled, as an evidence of good faith, to lend a hand in the construction of a communist barricade, sometimes wrangling with gate guards who retained an ignorant and superstitious reverence for pass-words and suchlike vain formalities, sometimes with his precious wagons deprived of their horses, or even upset into a ditch, by the imperious demands of artillery trains or of other purely military monopolists of the causeway. In spite of all difficulties, Sir John was always to be found where his presence and his help were most essential, supplying the surgeons with medicines, dressings, food, and other necessaries, as well as with the money required for making purchases of such things as could be obtained in the locality. After the close of the Franco-German war, he returned to France in charge of great gifts of seed to the ruined agriculturists, sharing the work of distribution with the late Mr. John Bellows and the other representatives of the Society of Friends, and thus taking the most effective possible steps for the repair of the devastation of which he had so shortly before been a witness.

On the outbreak of the South African war, Sir John was temporarily resting from his arduous labours, but he was recalled to activity by a request first that he would design a hospital train, the initiative of which was due to H.R.H. Princess Christian, and next that he would see it upon the rails in South Africa and fairly at work. The train, for the general design and internal arrangements of which Sir John was responsible, consisted of seven bogie carriages, four of which were constructed to carry 72 invalids, and 16 orderlies, whilst the others were arranged for two medical officers, two sisters, and other members of the staff, a dining-room, dispensary, kitchen, etc. Originally intended to be put on the rails at Cape Town, the train was diverted to Durban, and made its first trip, under Sir John's supervision, to Ladysmith, whence it safely brought back 54 of the worst cases in the garrison, and delivered them safely on board the hospital ship *German*. From this time Sir John, who had been induced to abandon his original intention of soon returning to England, and to remain in South Africa as Chief Commissioner of the Red Cross Society in succession to Colonel Young, was almost ubiquitous, and was everywhere helpful, but he declines to dwell with any minuteness upon the events of what he describes as recent history. It is at least certain that his own share in these events will never be forgotten either by the sick and wounded who derived benefit from his supreme power of organising and arranging, or by those who witnessed the way in which even red tape relaxed its folds before his energy and his persuasiveness.

